# The yield of endometrial aspiration in women with various risk factors and bleeding abnormalities

**DOI:** 10.1186/s40834-016-0020-7

**Published:** 2016-06-08

**Authors:** Anita L. Nelson, Lisa Vasquez, Roya Tabatabai, Samuel S. Im

**Affiliations:** 1 Los Angeles BioMedical Research Institute at Harbor-UCLA Medical Center, Torrance, CA USA; 2 Department of Obstetrics and Gynecology, Harbor-UCLA Medical Center, Torrance, CA USA; 3 Department Obstetrics and Gynecology, Healthcare Partners, Montebello, CA USA; 4 Department of Hematology and Oncology, Olive View-UCLA Medical Center, Sylmar, CA USA; 5 1457 3rd Street, Manhattan Beach, CA 90266 USA

**Keywords:** Abnormal uterine bleeding, Endometrial aspiration, Endometrial biopsy, Endometrial carcinoma, Endometrial hyperplasia with atypia

## Abstract

**Background:**

Even in the face of a substantial increase in the numbers of endometrial cancer cases and in the numbers of women who have risk factors, there is no clear agreement about the indications for assessing the endometria of women with abnormal bleeding or about the tools to use in that assessment. This study sought to determine in a group of high risk women with abnormal uterine bleeding, the probability that an outpatient endometrial aspiration would identify significant pathology.

**Methods:**

Retrospective cohort study of the histology from endometrial aspirations performed from 2001 to 2008 for abnormal uterine bleeding at Harbor-UCLA Medical Center and its satellite public health clinics. Medical records were reviewed in detail to assess risk factors, descriptions of bleeding abnormalities and histologic results.

**Results:**

The charts of 1601 women who underwent 1636 endometrial biopsies for a wide variety of abnormal uterine bleeding patterns yielded 73 (4.6 %) cases of endometrial carcinoma, 43 cases of atypical endometrial hyperplasia (2.7 %), for an overall yield of significant pathology of 7.2 %. Hyperplasia without atypia was found in another 83 cases (5.2 %). Obesity, diabetes and postmenopausal age are associated with an increased risk of significant pathology. Bleeding patterns were so poorly documented that analysis of yield by this factor should be viewed with caution.

**Conclusions:**

The probability of detecting significant uterine pathology is greatest among obese, diabetic postmenopausal women with diabetes (26.3 %). Conversely, the probability of identifying significant pathology in younger women without risk factors is less than 2 %. For women who perceive their individualized risk estimate to be too small to justify an endometrial biopsy, it may be possible to offer oral higher dose progestin therapy on the condition that persistent abnormal bleeding will require more intensive evaluation. These estimates of absolute risk of being diagnosed with significant pathology on endometrial biopsy may be helpful to patients as they consider giving informed consent for the procedure.

## Background

Endometrial carcinoma is the most frequent reproductive cancer in US women; the American Cancer Society estimates that 54,870 new cases will be diagnosed in 2015 [1]. Despite better understanding of the risk factors for the most common form of endometrial carcinoma, endometrial adenocarcinoma and its precursor lesion, endometrial intraepithelial neoplasia/atypical endometrial hyperplasia, no routine screening tests are currently available. On the other hand, diagnostic testing has evolved over the last few decades. Operative curettage was first replaced by office-based sampling [2–9]. Hysteroscopic evaluation and directed biopsy [10–12], ultrasonographic assessments [13–18], saline infusion sonography [12, 19–22], and saline infusion sonography-directed endometrial biopsy [22] have followed and are used in different settings to evaluate women with abnormal bleeding for endometrial pathology.

Endometrial evaluation has generally been recommended to evaluate “abnormal uterine bleeding” in at risk women [23, 24]. ACOG has recommended endometrial sampling for women as young as age 19–39 years who do not respond to medical therapy or who have prolonged periods of unopposed estrogen stimulation. Risk factors identified included nulliparity, hypertension, body mass index >30, irregular menstruation and family history [25]. Most recently ACOG has characterized suspicious bleeding as irregular menses, intermenstrual bleeding and postmenopausal bleeding and said that the decision to histologically evaluate abnormal uterine bleeding in premenopausal women should be based on symptomatology and clinical presentation [26, 27]. The Canadian Society of Obstetricians and Gynecologists recommends endometrial sampling in women presenting with abnormal uterine bleeding and age over 40 or weighing more than 90 kg [28]. In all of these cases, the definitions of “irregular”, “abnormal” and “heavy” bleeding are not standardized. Although there are multiple well established risk factors in the development of endometrial cancer including diabetes, unopposed estrogen stimulation and nulliparity [29], rarely are risk factors other than age and weight considered in the guidelines.

While much attention has been paid to the relative risks of developing endometrial cancer and to the sensitivity and specificity of these various testing modalities, one clinical question has not received as much attention: If a woman undergoes endometrial sampling, what is the probability that significant pathology will be detected to justify such invasive testing in lieu of other options, such as providing empiric progestin therapy? In the absence of consensus guidelines regarding when to perform endometrial biopsy, information regarding yield of such a procedure may assist providers and patients in making an informed decision.

Harbor-ULCA Medical Center in Torrance, California has traditionally provided care to indigent, uninsured women who have limited access to medical services. Endometrial biopsies have consistently been performed using Uterine Explora Model 1 (Cooper Surgical, Trumbull CT) with 3 mm OD plastic curette with cutting edges and a 10 cc removable syringe because it has been thought that such equipment would minimize the frequency of inadequate samples. The purpose of this study was to determine the probability of finding significant endometrial pathology (hyperplasia with atypia or endometrial carcinoma) on aspiration of women with different risk factors who present with what their clinicians categorize as “abnormal bleeding”. This information may guide clinicians in deciding when to recommend endometrial aspiration to individual patients and may provide patients a basis upon which to decide which to give or withhold informed consent for the procedure.

## Methods

Consent to conduct this research was obtained from the Research Committee and from the John F. Wolfe Human Subjects Committee of the Los Angeles BioMedical Research Institute at Harbor-ULCA Medical Center on an exempt basis because the study posed no more than minimal risk and because no personal identifying information was collected. Each patient’s chart was consulted for her age group, medical problems that might contribute to abnormal bleeding or affect her risk for having endometrial disease (including her height and weight), the dimensions of her endometrial specimen, and her histological results. An attempt was also made to extract information about the pattern of the woman’s abnormal bleeding (infrequent, prolonged, and/or heavy, intermenstrual, postcoital, etc.) to provide additional criteria to aid in decision-making. Cases were excluded if the endometrial aspiration was not done solely to evaluate bleeding abnormalities, e.g. it was as part of a work-up of abnormal cervical cytology. Yields are expressed as percent of samples that were found to have significant disease (endometrial atypia or carcinoma). Atypia was included in the category of significant disease since it has been shown to be premalignant and in up to 40 % of cases, there is an undetected adenocarcinoma [30]. Fishers two-tailed exact test was used to compare frequencies of outcomes; *p* < 0.05 was used to define statistically significant differences.

## Results

The paper charts and electronic medical records of 1601 women who presented with complaints of abnormal vaginal bleeding were reviewed. Important patient characteristics are displayed in Table 1. Perimenopausal women (age 41–50) constituted 48.3 % of the study population. Patient weights ranged from 41 to 219 kg; BMIs ranged from 17 to 83.9 kg/m^2^ (mean = 33.02 kg/m^2^). Most women (58.0 %) were obese and in 31 % of the cases, the woman’s weight exceeded 90 kg. Leiomyoma were reported in 29.8 %. In 14.7 % cases, a history of diabetes was recorded; 5.3 % had thyroid dysfunction; 1.9 % provided a history of breast cancer and 1.1 % volunteered a history of prior endometrial hyperplasia. Hemoglobin test results were reported in 78.6 % of cases; those hemoglobin levels ranged from 2.1 to 16.4 g/dL and 13.8 % of women had severe anemia (hemoglobin <8 g/dL). Pulse rates ranged from 40 to 135 beats per minute.

**Table 1:** Characteristics of study population by age group

Age group (years)	Number endometrial biopsies	Mean BMI^a^(kg/m^2^)	Percent overweight (%)	Percent obese (%)	Percent diabetes (%)	Percent with leiomyoma (%)
<21	5	33.48	0	75.0	0	0
21–30	88	39.83	15.7	82.0	7.9	9.0
31–40	317	34.49	30.3	58.9	11.1	30.9
41–50	789	31.57	27.1	53.8	12.4	37.2
51–55	232	32.56	24.7	60.0	18.1	27.7
56–55	173	34.25	25.4	60.4	32.0	17.8
>65	31	32.07	16.7	53.3	26.6	−10
Total	1,635	33.03	26.3	58.0	15.0	30.4

^a^ *BMI* body mass index

For the study population as a whole, 27 cases of atypical hyperplasia and 74 cases of uterine (non-cervical) carcinoma were diagnosed. Table 2 demonstrates the effect obesity had on the yield of finding significant pathology. While the incidence of carcinoma did not differ significantly by BMI (≤30 vs > 30 kg/m_2_) overall (4.0 % vs 4.9 %) (*p* = 0.4); the incidence of atypical hyperplasia appeared more frequently in obese women (0.7 % vs 2.4 %) (*p* = 0.02). The impact of obesity was greatest in the finding of simple hyperplasia (2.2 % vs 7.3 %) (*p* < 0.0001).

**Table 2:** Yield of endometrial aspiration: impact of obesity

Pathology result	Total population		BMI ≤ 30*	BMI >30*
	Number	%	%	%
Inadequate/limited	206	12.6	14.1	11.5
Endometritis	172	10.5	12.3	9.5
Proliferative/disordered proliferation	444	27.2	26.9	27.6
Secretory/decidualized/menstrual	351	21.5	24.4	20.6
Atrophy	97	5.9	6.7	4.8
Polyp	158	9.7	8.1	10.6
Simple hyperplasia	89	5.4	2.2	7.3
Complex hyperplasia	16	1.0	0.6	1.3
Hyperplasia with atypia	27	1.6	0.7	2.7
Endometrial carcinoma	74	4.5	4.1	4.9
Total	1635	99.9	100.1	100.4

* BMI body mass index in Kg/m^2^

However, when age is considered a slightly different picture emerges. Table 3 displays the percent of the endometrial biopsies found to have atypia or carcinoma by age and BMI groups. Again it can be seen that these abnormalities were more frequent in obese women at every age, but weight’s influence was greatest in the younger women.

**Table 3:** Percent of biopsies demonstrating hyperplasia with atypia or carcinoma by age and BMI groups

BMI	Age (years)			
	<40	41–51	>50	All
≤30	2.1 %	1.9 %	12.9 %	4.8 %
>30	5.9 %	4.0 %	14.1 %	7.3 %

*BMI* body mass index in Kg/m^2^

Table 4 displays histological findings reported by age groups for obese and non-obese diabetic vs. non-diabetic women. In every age group with sufficient numbers, women with diabetes had higher rates of significant disease (atypia or carcinoma) than those in whom diabetes had not previously been diagnosed. The highest yield (22.4 %) was seen in postmenopausal women with BMI ≥ 30 kg/m^2^ and diabetes. The prevalence of endometritis was consistently higher among younger women compared to postmenopausal women, but the frequency of atrophic findings were reversed in those two groups. Despite the routine use of large plastic aspirator with a cutting edge, one eighth (12.58 %) of the specimens were reported to be “inadequate” or “of limited value”. Rates varied slightly by age, women ≤ 50 years (11.2 %) versus > 50 years (17.4 %) (*p* = 0.0017). These devices were designed to facilitate biopsy in the face of active bleeding. In this study, 59.7 % of women were reported to have no bleeding; heavy or moderate bleeding was seen in 17.7 % of patients. Volumes of specimens varied significantly; in the 1464 cases where three dimensions of the specimen were provided by the pathologist, the mean volume was 2.9 mm^3^, but the standard deviation was 7.4; the median volume was 1.0 mm^3^.

**Table 4:** Histology by age, BMI and diabetes status (%)

	Age <40 years				Age 41–50 years				Age > 50 years			
	BMI <30		BMI ≥30		BMI <30		BMI ≥30		BMI <30		BMI ≥30	
	No DM	DM	No DM	DM	No DM	DM	No DM	DM	No DM	DM	No DM	DM
Number of subjects	132	11	226	30	334	23	347	73	146	24	179	76
Inadequate/limited	12.9	27.3	10.2	10.0	12.6	8.7	8.9	11.1	15.8	29.2	16.2	17.1
Endometritis	12.9	18.2	10.2	6.7	14.4	13.0	12.7	11.1	6.8	8.3	3.9	5.3
Proliferative/disordered	28.0	36.4	29.6	13.3	30.5	26.1	32.3	24.7	18.5	16.7	27.4	9.2
Secretory/decidualized/menstrual	29.5	18.2	24.3	10.0	29.0	39.1	26.2	24.7	11.0	--	6.1	7.9
Atrophy	0	0	0	0	0.8	4.3	1.4	2.7	23.3	29.2	16.8	14.5
Polyp	12.1	0	8.8	23.3	7.2	8.7	12.2	8.2	8.2	--	11.2	7.9
Simple hyperplasia	2.3	0	11.1	16.7	2.7		3.7	5.5	2.1	--	7.8	9.2
Complex hyperplasia	0	0	0.9	6.7	0.3		0	4.1	2.1	--	1.7	2.6
Hyperplasia with atypia	0.8	0	2.2	6.7	0.8		2.3	4.1	0.7	--	0.6	3.9
Endometrial carcinoma	1.5	0	2.7	6.7	1.2		0.9	4.1	11.6	16.7	8.4	22.4

*BMI* body mass index in Kg/m^2^, *DM* diabetes melitis

Endocervical curettage was performed in conjunction with the endometrial aspiration in 1397 cases. Cervical dysplasia was detected in 6 women and cervical carcinoma was diagnosed in 29 women, but in 8 cases those women were found to have adenocarcinoma of the cervix in conjunction with endometrial carcinoma. Therefore, only an additional 21 cervical cancers were detected only by endocervical curettage.

Table 5 displays the categories of bleeding abnormalities that prompted the aspiration (when such data could be gleaned from for the medical records) by age group and BMI group. The most common patterns were intermittent, prolonged or excessive bleeding, followed by postmenopausal bleeding.

**Table 5:** Bleeding or ultrasound abnormalities which prompted biopsy (number specimens)

Bleeding or ultrasound abnormality	Age ≤ 40 years		Age 41–50 years		Age ≥50 years		Total
	BMI <30	BMI ≥30	BMI ≤30	BMI >30	BMI ≤30	BMI >30	
Infrequent bleeding	20	85	50	23	6	15	199
Prolonged or excessive multiple episodes	75	110	232	212	24	44	697
Single episode	31	55	71	68	10	9	244
Intermenstrual	18	21	40	37	13	7	136
Postmenopausal	1	1	28	16	108	170	324
Post-coital	4	6	5	6	3	1	24
Thickened endometrium (ultrasound)	3	2	4	4	10	12	35
Unclassified	--	--		1	1	--	5
Total	142	255	415	356	170	255	1593

*BMI* body mass in Kg/m^2^

Table 6 displays the histological outcomes of the endometrial biopsies done to evaluate women with different bleeding patterns. Postmenopausal bleeding was associated with the highest yield of significant disease (atypia or carcinoma) (16.7 %) and inadequate or limited results (17.7 %), polyps (10.7 %) and, atrophy (22.7 %). At the other extreme, women of any age who had normal menstrual intervals, but prolonged or excessive bleeding had only a 1.3 % change of significant disease. A single episode of heavy or prolonged bleeding was associated with atypia or carcinoma in only 2.4 % of women of all ages. Curiously, the bleeding patterns had little predictive influence for endocervical samples (Table 7). None of the women diagnosed with cervical dysplasia or carcinoma complained of postcoital bleeding. Only “inflammatory changes” (4.3 %) or “unremarkable endocervical mucosa” (95.7 %) were reported with postcoital bleeding.

**Table 6:** Histologic results by bleeding or ultrasound abnormality category (%)

Histology	Infrequent bleeding	Prolonged or excessive multiple episodes	Prolonged or excessive single episode	Intermenstrual	Postmenopausal	Postcoital	Thickened endometrium	Unclassifiable
Infrequent/limited	12.8	12.0	6.2	13.6	17.0	4.2	23.3	0
Endometritis	5.3	13.7	14.2	12.1	3.3	16.7	6.7	0
Proliferative/disordered proliferation	33.7	30.6	25.7	22.9	19.1	41.7	13.3	100
Secretory/decidualized/menstrual	20.3	27.3	32.8	22.9	3.3	8.3	10.0	0
Atrophy	1.1	0.9	--	2.1	22.7	4.2	26.7	0
Polyploid	11.2	8.3	11.2	10.7	10.7	12.5	6.7	0
Simple hyperplasia	8.0	4.5	6.2	4.3	6.0	4.2	6.7	0
Complex hyperplasia	1.6	0.6	0.8	0.7	1.2	0	6.7	0
Atypia	2.7	1.0	1.2	2.9	2.4	8.3	0	0
Endometrial carcinoma	3.2	1.0	0.8	7.9	14.3	0	0	0
Total	99.9	100.1	99.8	100.1	100.0	100.1	100.1	100.0

**Table 7:** Endocervical curettage histologic results by bleeding abnormalities (%)

Histologic finding	Infrequent bleeding	Prolonged or excessive multiple episodes	Prolonged or excessive single episode	Intermenstrual	Postmenopausal	Postcoital	Thickened endometrium	Unclassifiable
Infrequent/limited	5.6	4.1	5.3	5.8	3.2	0	4.8	100
Unremarkable endocervical mucosa	88.1	86.6	84.2	82.5	85.4	91.3	71.4	0
Inflammation/infection	3.8	6.4	7.7	8.3	6.1	4.3	23.8	0
Polyp	0.6	1.5	1.4	0.8	0.7	0	0	0
Dysplasia	0.6	0.9	0.5	1.7	1.1	0	0	0
Cervical cancer: squamous	0	0.2	0	--	1.8	0	0	0
Cervical cancer: endocervical	1.3	0.3	1.0	0.8	1.8	0	0	0
Total ECC	100.0	100.0	100.1	99.9	100.1	100.1	100.0	100.0

### Discussion and Conclusions

The sensitivities and specificities of diagnostic tests have been compared to a variety of standards, including hysterectomy specimens [6, 20, 31–33], operative dilation and curettage [32, 34, 35], hysteroscopically directed biopsy/curettage [10, 11, 21, 33], and ultrasound-directed biopsy/curettage [15, 20, 21, 36]. Studies have also evaluated the influence of age [37–40], weight [37–43], diabetes [44, 45], and cycle frequency [38, 42, 46] as risk factors for carcinoma or hyperplasia in both premenopausal and postmenopausal women. Our study sought to answer a slightly different clinical question: What is the likelihood that a woman who presents with a bleeding abnormality with a given BMI and age would have a significant disease (endometrial intraepithelial neoplasia atypical hyperplasia or carcinoma) detected by one endometrial aspiration?

The women in our study had among the highest prevalence or of endometrial cancer reported to date, which allows us to provide a more secure estimate of the upper limit of the potential for disease detected by endometrial aspiration [46]. None of the women in this study was using unopposed estrogen therapy. However, the majority had BMI >30 kg/m^2^. Obesity is a well-recognized as a risk factor for endometrial cancer with a 200–400 % linear increase in risk with each point increase in BMI [47], but there is some controversy about whether obesity is more impactful among premenopausal women [35] or among postmenopausal women [40]. We found that BMI increased the risk of atypia in every age group, but carcinoma detection rates were similar in obese and non-obese women. Postcoital bleeding was not associated with cervical dysplasia or carcinoma, but given the small number of women with that complaint, this finding may not contradict the finding of Rosenthal et al. that invasive cancer is rare in women with postcoital bleeding, but more common than in the general population [48].

The premenopausal women in our study had higher risk features than earlier studies and higher rates of detection of endometrial hyperplasia (with or without atypia) and endometrial cancer, (8.8 %). Previous Investigators have reported rates of combined hyperplasia and cancer on endometrial biopsy of women presenting with abnormal bleeding ranging from 5 to 7.7 % [37, 38]. The fact that our estimate of risk for all forms of pathology exceed previously reported finding supports their value as upper limits of risk.

The impact that age has on the prevalence of significant pathology had been reported in a large study of pathology reports. Again women of that age group in our study had proportionately more cases of pathology. Because we had more information about the women than earlier investigators, we were able to isolate the impacts of weight as well as known diabetes might have on the probability that significant pathology would be identified on biopsy [49].

Polyps were diagnosed on pathology in 12.1 % of our subjects overall. Malignancy confined to the polyp was reported in only one case, which is much lower than the 4.2 % rate of malignancy or atypia found within polyps reported earlier in symptomatic postmenopausal women [50]. Because we specifically excluded women who had abnormal cervical cytology, we cannot comment on the utility of HPV testing to distinguish between cervical and endometrial cancer in women with atypical glandular changes as recommended by Castle et al. [51].

In patients with abnormal bleeding related to obesity, thyroid dysfunction, etc., a short course of hormonal therapy for abnormal of bleeding will not be curative as it would not treat the underlying condition [52]. However, prolonged therapy with progestin will prevent development of endometrial hyperplasia and control bleeding excesses until the underlying cause is addressed [53]. Progestin containing OCPs and progestin-releasing IUDs are effective in treating endometrial hyperplasia via progestin-meditated reversal of the PTEN suppressor genes [54, 55]. The relatively low rates of significant pathology found in our study among the younger women with normal risk factors and perimenopausal women with single episodes of heavy bleeding may make those women hesitate to agree to biopsy, but to be more accepting of a therapeutic trial of progestin therapy with the understanding that persistent abnormal bleeding would change these estimates and indicate a stronger need for biopsy. The information provided by this analusis may also be helpful in calculating the cost effectiveness of screening women with different clinical presentations [40, 56].

Torres et al. reported that one fourth of patients diagnosed with endometrial cancer had previous benign endometrial sample results [57]. Close follow-up is essential as are measures to prevent recurrent abnormal bleeding or development of hyperplasia, but these services often not implemented [53, 58–65]. Over 1 % of patients in our study presented with a history of prior endometrial hyperplasia, but none was on any suppressive therapy. This is not an isolated problem, in one series of women treated for hemoglobin ≤5 mg/dL, due to excessive vaginal bleeding, over 25 % had been previously transfused, but none of these women was on any medical therapy to control future menstrual losses [66].

Despite the large number of cases studied and the high prevalence of disease found in our study, our results are not precise. However, they can provide estimates of the upper limit of finding significant pathology with endometrial biopsy. We have analyzed the data in various combinations of risk factors to provide the most information about the absolute risk, not relative risk. We offer our data to others with interest in this area in hopes that it may be combined with data from other investigators to refine the estimates of the yield of endometrial sampling in different clinical settings [67]. However, we recognize that these data are not applicable for one of the most common clinical scenarios—bleeding in women using postmenopausal hormone therapy—since none of our subjects reported current use of those agents.

As a retrospective study, we are not able to characterize the bleeding patterns with as much accuracy as would be possible in a prospective trial [68]. Accurate categorization into bleeding groups was challenging given the paucity of menstrual data recorded in some of the records. However, at a minimum, it is hoped that our detailed analysis by age group, BMI and presenting bleeding pattern may help provide more individually relevant information for the clinician and the patient to utilize when making diagnostic decisions.

### Condensation

The probability of finding significant uterine pathology on endometrial aspiration varies greatly by patient risk factors and clinical presentation, but is generally less than 2 % in premenopausal women with BMI <30 kg/m^2^ and without diabetes who complain of abnormal bleeding. The highest yield (22.4 %) was in postmenopausal women with diabetes and BMI ≥30 kg/m^2^.
